# Comparative efficacy and safety of bortezomib, thalidomide, and dexamethasone (VTd) without and with daratumumab (D‐VTd) in CASSIOPEIA versus VTd in PETHEMA/GEM in transplant‐eligible patients with newly diagnosed multiple myeloma, using propensity score matching

**DOI:** 10.1002/jha2.129

**Published:** 2020-11-07

**Authors:** Philippe Moreau, Cyrille Hulin, Sonja Zweegman, Mahmoud Hashim, Yannan Hu, Bart Heeg, Carla de Boer, Veronique Vanquickelberghe, Tobias Kampfenkel, Jianming He, Annette Lam, Sarah Cote, Pieter Sonneveld

**Affiliations:** ^1^ Nantes University Hospital Hôtel‐Dieu Nantes France; ^2^ Hospital Center University De Bordeaux Bordeaux France; ^3^ Amsterdam UMC, Vrije Universiteit Amsterdam Cancer Center Amsterdam Amsterdam The Netherlands; ^4^ Ingress Health Rotterdam The Netherlands; ^5^ Janssen Research & Development Leiden The Netherlands; ^6^ Janssen Research & Development Beerse Belgium; ^7^ Janssen Global Services LLC Raritan New Jersey USA; ^8^ Erasmus MC Cancer Institute Rotterdam The Netherlands

**Keywords:** clinical trials, monoclonal antibodies, multiple myeloma, thalidomide

## Abstract

**Background:**

Traditional bortezomib, thalidomide, and dexamethasone (VTd) regimens for patients with newly diagnosed multiple myeloma (NDMM) include doses of thalidomide up to 200 mg/day (VTd‐label). Clinical practice has evolved to use a lower dose (100 mg/day) to reduce toxicity (VTd‐mod), which was evaluated in the phase III CASSIOPEIA study, without or with daratumumab (D‐VTd; an anti‐CD38 monoclonal antibody). We used propensity score matching to compare efficacy and safety for VTd‐mod and D‐VTd with VTd‐label.

**Methods:**

Patient‐level data for VTd‐mod and D‐VTd from CASSIOPEIA (NCT02541383) and data for VTd‐label from the PETHEMA/GEM study (NCT00461747) were analyzed. Propensity scores were estimated using logistic regression, and nearest‐neighbor matching procedure was used. Outcomes included overall survival (OS), progression‐free survival (PFS), time to progression (TTP), postinduction and posttransplant responses, as well as rate of treatment discontinuation and grade 3/4 peripheral neuropathy.

**Results:**

VTd‐mod was noninferior to VTd‐label for OS, PFS, TTP, postinduction very good partial response or better (≥VGPR) and overall response rate (ORR). VTd‐mod was significantly better for posttransplant ≥VGPR and ORR versus VTd‐label. VTd‐mod safety was not superior to VTd‐label despite the lower thalidomide dose. D‐VTd was significantly better than VTd‐label for OS, PFS, TTP, postinduction and posttransplant ≥VGPR and ORR, and was noninferior to VTd‐label for safety outcomes.

**Conclusions:**

In transplant‐eligible patients with NDMM, D‐VTd had superior efficacy compared with VTd‐label. Despite a lower dose of thalidomide, VTd‐mod was noninferior to VTd‐label for safety and was significantly better for posttransplant ≥VGPR/ORR. These data further support the first‐line use of daratumumab plus VTd.

## 1 INTRODUCTION

The triple‐drug combination of bortezomib, thalidomide, and dexamethasone (VTd) followed by autologous stem‐cell transplantation (ASCT) is a standard of care for the treatment of patients with newly diagnosed multiple myeloma (NDMM) who are transplant‐eligible [1]. The traditional dosing schedule for pretransplant induction therapy is based on an escalating dose of thalidomide per the product label (VTd‐label) and comprises a 28‐day cycle of bortezomib (1.3 mg/m^2^ subcutaneously on days 1, 8, 15, and 22), thalidomide (100 mg orally on days 1–21), and dexamethasone (20 mg on the day of and the day after bortezomib dosing, or 40 mg on days 1, 8, 15, and 22), typically repeated for up to six cycles [1, 2].

As higher doses of thalidomide have been associated with peripheral neuropathy [3], clinical practice has evolved to use a modified version of VTd (VTd‐mod), which features a lower dose of thalidomide (100 mg daily) to potentially reduce toxicity. This dosing regimen recently gained approval in the United States, Europe, and Brazil in combination with daratumumab [4, 5, 6], a human monoclonal antibody targeting CD38 that has an immunomodulatory mechanism of action. Approval was based on the results of the phase III CASSIOPEIA trial Part 1 in transplant‐eligible patients with NDMM [7]. The dosing regimen in CASSIOPEIA Part 1 comprised four 28‐day cycles of pre‐ASCT induction therapy and two 28‐day cycles of post‐ASCT consolidation therapy with bortezomib, thalidomide (100 mg daily), and dexamethasone, without or with daratumumab (D‐VTd). Treatment with D‐VTd improved the depth of response and progression‐free survival (PFS) in patients with NDMM [7]. Part 2 of this study, which is investigating daratumumab monotherapy maintenance (16 mg/kg every 8 weeks until progression, or for a maximum of 2 years) versus observation in patients who achieved a partial response or better, is ongoing.

To date, there have been no randomized clinical trials (RCTs) that directly compare the efficacy and safety of VTd‐mod or D‐VTd versus VTd‐label. It is difficult to draw meaningful conclusions from indirect comparisons between published aggregated clinical trial data because unadjusted comparisons of outcomes are prone to confounding bias, due to variation in patient characteristics between treatment populations. However, statistical methods that control for differences in baseline covariates, such as propensity score matching (PSM), can be utilized to estimate differences between treatment regimens in the absence of a head‐to‐head comparison [8, 9]. The objective of the current PSM analysis was to compare the efficacy and safety of the VTd‐mod and D‐VTd regimens versus VTd‐label in patients with NDMM who are transplant eligible.

## 2 METHODS

### 2.1 Data sources

Data for the PSM were from two phase III clinical trials, PETHEMA/GEM (ClinicalTrials.gov, NCT00461747), and CASSIOPEIA (ClinicalTrials.gov, NCT02541383).

Data for the VTd‐label group were taken from the PETHEMA/GEM study, in which patients were randomized to one of three regimens: the alternating chemotherapy regimens vincristine/carmustine/melphalan/cyclophosphamide/prednisone and vincristine/carmustine/doxorubicin/dexamethasone, followed by bortezomib; vs thalidomide/dexamethasone; vs VTd [10, 11]. Patients in the VTd group received pre‐ASCT induction therapy (six 28‐day cycles) with bortezomib 1.3 mg/m^2^ on days 1, 4, 8, and 11; thalidomide (Cycle 1 escalating doses up to 50 mg on days 1‐14 and 100 mg on days 15‐28, then 200 mg thereafter); and oral dexamethasone 40 mg on days 1‐4 and 9‐12. This regimen was followed by post‐ASCT maintenance (up to 3 years) of subcutaneous (SC) interferon alfa‐2b 3 MU three times/week; and oral thalidomide 100 mg/day or 100 mg/day with bortezomib, 1 cycle every 3 months.

Data for the VTd‐mod and D‐VTd groups were taken from the CASSIOPEIA study, a two‐part, open‐label study conducted in patients with NDMM who were transplant‐eligible [7]. In Part 1, patients were randomized 1:1 to pre‐ASCT induction therapy (four 28‐day cycles) with either VTd‐mod or VTd‐mod with daratumumab (D‐VTd), followed by post‐ASCT consolidation therapy (two 28‐day cycles) of VTd‐mod or D‐VTd. Patients received intravenous (IV) daratumumab 16 mg/kg once weekly in Cycles 1‐2 and every 2 weeks in Cycles 3‐4; SC bortezomib 1.3 mg/m^2^ on days 1, 4, 8, and 11; oral thalidomide 100 mg/day; and oral or IV dexamethasone 40 mg on days 1, 2, 8, 9, 15, 16, 22, and 23 at Cycles 1‐2 followed by 40 mg on days 1 and 2 and 20 mg on days 8, 9, 15, and 16 of Cycles 3‐4. Part 2 of this study is ongoing. Patients who achieved partial response or better at day 100 posttransplant were rerandomized to observation or daratumumab maintenance (16 mg/kg, monotherapy) every 8 weeks for a maximum of 2 years or until disease progression.

The CASSIOPEIA and PETHEMA/GEM study designs are summarized in Additional file 1 (in the Supporting Information), and efficacy outcomes for both studies are summarized in Additional file 2 (in the Supporting Information) [7, 10, 11]. Individual patient‐level data were obtained from the sponsor for the PETHEMA/GEM (VTd‐label) and CASSIOPEIA (VTd‐mod and D‐VTd) trials; data were validated with their respective clinical study reports. The PETHEMA/GEM study was approved by the Spanish National Health Service and by all the local institutional ethics committees and was conducted in accordance with the principles of the Declaration of Helsinki. The CASSIOPEIA study was done in accordance with the principles of the Declaration of Helsinki and the International Conference on Harmonisation Good Clinical Practice guidelines. All patients gave written informed consent.

### 2.2 Propensity score matching

PSM was used to correct for differences in baseline characteristics in the CASSIOPEIA and PETHEMA/GEM trials. The National Institute for Health and Care Excellence [NICE] decision tree was used to determine which propensity score methodology best suited the data for this analysis [12]. As there was some imbalance in baseline characteristics before matching, and good balance was possible to achieve after matching, analysis on matched samples was deemed more appropriate in both the primary and sensitivity analyses.

In an exploratory analysis, several types of matching methods were applied to pick the best performing method. For each method, the distribution of propensity scores before and after matching and the postmatch balance between treatment groups (VTd‐mod vs VTd‐label or D‐VTd vs VTd‐label) was assessed. To determine how adequately PSM balanced the covariates, pre‐ and postmatch balance between treatment groups (VTd‐mod vs VTd‐label or D‐VTd vs VTd‐label) was assessed using standardized mean differences for the included covariates (described below), with values >0.1 suggesting potentially important imbalances [13]. Additionally, chi‐square tests were performed to assess the statistical significance of differences in covariates between treatment groups before and after matching. This assessment determined that the best performing PSM method was nearest‐neighbor matching (without replacement). A 2:1 ratio was used (number of VTd‐mod or D‐VTd patients matched to each VTd‐label patient). Propensity scores were estimated using logistic regression, and matching was carried out using the Matchit R package [14].

Propensity score distribution in both treatment groups was assessed before and after matching to assess the degree of overlap. Additionally, propensity score distributions in matched and unmatched patients were assessed to determine whether the individuals not matched were in some specific part of the propensity score continuum. After excluding unmatched samples, outcomes observed in the matched sample were compared directly using a suitable measure of treatment effect for different endpoints.

### 2.3 Covariates

The following covariates were identified for matching (based on expert opinion): age, gender, Eastern Cooperative Oncology Group performance status, myeloma type, International Staging System (ISS), creatinine clearance, hemoglobin level, and platelet count. A sensitivity analysis included the same covariates as the primary analysis, plus cytogenetic risk. At baseline, the proportion of patients for whom cytogenetic testing was not done was higher in the PETHEMA/GEM study (37%) compared with the CASSIOPEIA study (<1%); therefore, patients for whom there was no cytogenetic testing were excluded from the sensitivity analysis.

### 2.4 Analysis variables and statistical methodology

The efficacy endpoints included in the PSM analysis were overall survival (OS), PFS, time to progression (TTP), overall response rate (ORR), complete response or better (≥CR), and very good partial response or better (≥VGPR) postinduction and posttransplant. In CASSIOPEIA, patients with confirmed daratumumab interference on serum M‐protein electrophoresis and immunofixation but who demonstrated all other CR criteria were considered to have CR [7]. Safety endpoints included in the analysis (for the induction phase only) were treatment discontinuation due to any grade of adverse events (AEs), treatment discontinuation due to grade 3 or 4 AEs, and grade 3 or 4 peripheral neuropathy.

For time‐to‐event outcomes (OS and PFS), hazard ratios (HRs) with two‐sided 95% confidence intervals (CIs) were estimated using stratified Cox regression models, fitted with treatment arm; *P* values for HRs and Kaplan‐Meier curves were based on the Wald test and log‐rank test, respectively. Comparison of HRs between treatment groups was reported with point estimates and 95% CIs. Response rates and AEs were analyzed based on an odds ratio calculated using a two‐sided 95% CI by fitting a logistic regression model. Results that did not achieve statistical significance (5%) were interpreted with the use of noninferiority margins [15, 16]. A targeted literature review identified noninferiority margins for response, safety, PFS, and OS as follows: 13% (rate difference), 13% (rate difference), 1.333 (HR), and 1.298 (HR), respectively [15]. Results that did not achieve significance and did not qualify per the noninferiority criteria were treated as inconclusive.

## 3 RESULTS

### 3.1 Patients, treatments, and baseline characteristics

The median duration of follow‐up was 35.9 months for VTd‐label and 18.8 months for VTd‐mod and D‐VTd. A total of 542 patients received VTd‐mod, 543 received D‐VTd, and 130 received VTd‐label. After matching, 250 patients for VTd‐mod and D‐VTd, as well as 125 patients for VTd‐label, were included in the analyses. The mean cumulative dose of bortezomib was lower in CASSIOPEIA than in PETHEMA/GEM (19.16 ± 2.93 mg/m^2^ vs 26.94 ± 6.89 mg/m^2^, respectively), as was the mean cumulative dose of thalidomide (9881 ± 2147 mg vs 20 730 ± 8949 mg).

Baseline characteristics for the efficacy analyses (both primary and sensitivity) before and after matching VTd‐mod to VTd‐label, and D‐VTd to VTd‐label, are summarized in Tables 1 and 2. Before matching for the VTd‐mod versus VTd‐label comparison, there were some potentially important imbalances among baseline characteristics, including age, ISS, creatinine clearance rate, hemoglobin level, platelet count, and cytogenetic risk. For the D‐VTd versus VTd‐label comparison, there were potential imbalances in age, ISS staging, creatinine clearance rate, platelet count, and cytogenetic risk at baseline. After matching for both comparisons, the groups were balanced on all covariates of interest (all estimated standardized mean differences were <0.1, and chi‐square test results were nonsignificant [*P *> .05]). Therefore, a comparison of outcomes on the matched sample was warranted.

**TABLE 1:** Key baseline characteristics for VTd‐mod (CASSIOPEIA) [7] and VTd‐label (PETHEMA/GEM) [10, 11] efficacy analyses, pre‐ and postmatching

				Matched patient population					
	Unmatched patient population			Primary analysis			Sensitivity analysis		
Variables	VTd‐mod, CASSIOPEIA	VTd‐label, PETHEMA/ GEM	Absolute standardized mean difference prematch	VTd‐mod, CASSIOPEIA	VTd‐label, PETHEMA/ GEM	Absolute standardized mean difference postmatch	VTd‐mod, CASSIOPEIA	VTd‐label, PETHEMA/ GEM	Absolute standardized mean difference postmatch
Sample size, *n*	542	130	NA	250	125	NA	156	78	NA
Age, mean, y	56.5	55.6	**0.120**	55.6	55.4	0.017	55.0	54.9	0.012
Male sex, %	58.9	58.5	0.008	60.8	57.6	0.065	56.4	59.0	0.052
ECOG PS ≥1, %	52.6	56.2	0.072	52.8	54.4	0.032	55.8	57.7	0.039
IgG myeloma, %	61.4	66.2	0.098	68.8	66.4	0.051	66.7	69.2	0.055
ISS staging, %									
ISS I	42.1	33.9	**0.218**	32.0	34.4	0.081	30.8	32.1	0.030
ISS II	43.0	43.9	NA	46.4	42.4	NA	46.2	44.9	NA
Creatinine clearance, mean, mL/min	100.1	86.5	**0.385**	89.0	86.8	0.070	88.5	89.4	0.029
Hemoglobin level, mean, g/L	114.8	111.2	**0.203**	111.5	111.5	0.003	108.8	109.6	0.050
Platelet count, mean, x10^9^/L	252.8	235.9	**0.191**	236.1	235.9	0.003	247.9	242.2	0.059
Cytogenetic risk, %									
Testing not done	0.4	36.9	**1.068**	Not included in the primary analysis	NA^a^	NA^a^	NA^a^		
High risk	15.9	12.3	NA		18.0	19.2	0.033		
Standard risk	83.8	50.8	NA		82.1	80.8	NA		

Standardized mean differences > 0.1 suggest potentially important imbalances (as indicated in bold).

Abbreviations: ECOG PS, Eastern Cooperative Oncology Group performance status; IgG, immunoglobulin G; ISS, Multiple Myeloma International Staging System; NA, not applicable; VTd, bortezomib, thalidomide, and dexamethasone.

^a^ In the sensitivity analysis, patients with no cytogenetic test done were excluded from the dataset.

**TABLE 2:** Key baseline characteristics for D‐VTd (CASSIOPEIA) [7] and VTd‐label (PETHEMA/GEM) [10, 11] efficacy analyses, pre‐ and postmatching

				Matched patient population					
	Unmatched patient population			Primary analysis			Sensitivity analysis		
Variables	D‐VTd, CASSIOPEIA	VTd‐label, PETHEMA/GEM	Absolute standardized mean difference prematch	D‐VTd, CASSIOPEIA	VTd‐label, PETHEMA/GEM	Absolute standardized mean difference postmatch	D‐VTd, CASSIOPEIA	VTd‐label, PETHEMA/GEM	Absolute standardized mean difference postmatch
Sample size, *n*	543	130	NA	250	125	NA	156	78	NA
Age, mean, y	56.8	55.6	**0.162**	55.3	55.4	0.025	55.0	54.9	0.004
Male sex, %	58.2	58.5	0.005	56.8	57.6	0.016	61.5	59.0	0.052
ECOG PS ≥1, %	51.2	56.2	0.100	54.0	54.4	0.008	56.4	57.7	0.026
IgG myeloma, %	64.6	66.2	0.032	64.0	66.4	0.050	69.2	69.2	<0.0001
ISS staging, %									
ISS I	37.6	33.9	**0.176**	33.2	34.4	0.040	32.7	32.1	0.041
ISS II	47.0	43.9	NA	44.4	42.4	NA	43.0	44.9	NA
Creatinine clearance (mean mL/min)	103.4	86.47	**0.359**	88.49	86.8	0.053	90.36	89.41	0.029
Hemoglobin level, mean (g/L)	112.4	111.2	0.071	111.2	111.5	0.013	111.6	109.6	0.107
Platelet count, mean (×10^9^/L)	248.7	235.9	**0.137**	240.7	235.9	0.053	243.9	242.2	0.018
Cytogenetic risk (%)									
Testing not done	0.2	36.9	**1.080**	Not included in the primary analysis	NA^a^	NA^a^	NA^a^		
High risk	15.1	12.3	NA		19.9	19.2	0.016		
Standard risk	84.7	50.8	NA		80.1	80.8	NA		

Standardized mean differences >0.1 suggest potentially important imbalances (as indicated by bold font).

Abbreviations: D‐VTd, bortezomib, thalidomide, and dexamethasone plus daratumumab; ECOG, Eastern Cooperative Oncology Group performance status; IgG, immunoglobulin G; ISS, Multiple Myeloma International Staging System; NA, not applicable; VTd, bortezomib, thalidomide, and dexamethasone.

^a^ In the sensitivity analysis, patients with no cytogenetic test done were excluded from the dataset.

### 3.2 Efficacy outcomes

**FIGURE 1:**
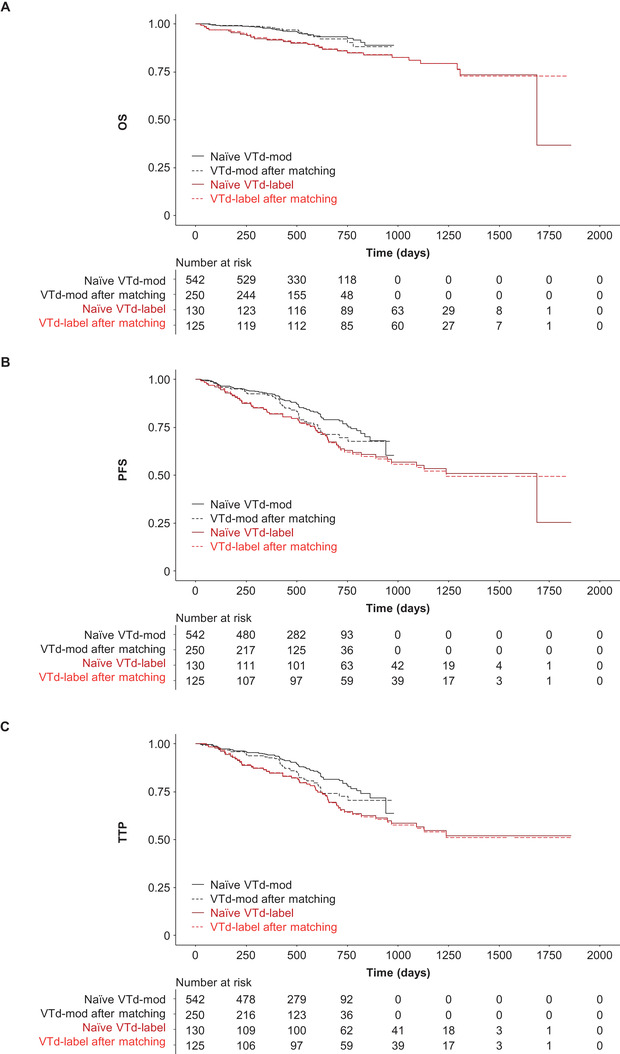
Naïve and matched comparisons of (A) OS, (B) PFS, and (C) TTP for VTd‐mod vs VTd‐label. Abbreviations: OS, overall survival; PFS, progression‐free survival; TTP, time to progression; VTd, bortezomib, thalidomide, and dexamethasone; VTd‐label, bortezomib/thalidomide/dexamethasone administered according to product labeling; VTd‐mod, bortezomib/thalidomide/dexamethasone modified dose

**FIGURE 2:**
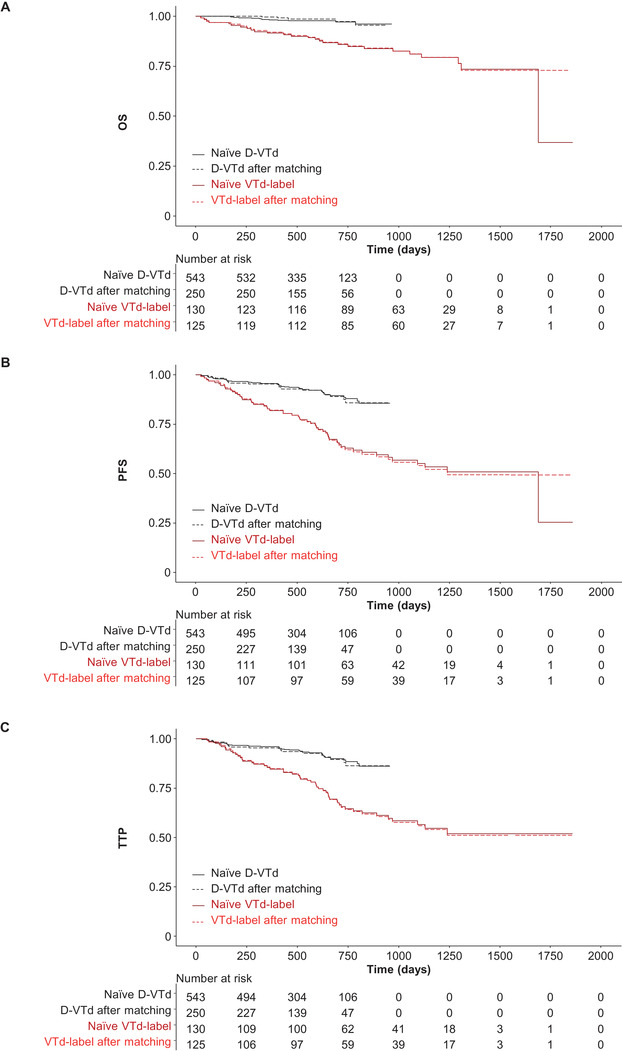
Naïve and matched comparisons of (A) OS, (B) PFS, and (C) TTP for D‐VTd vs VTd‐label. Abbreviations: D‐VTd, bortezomib, thalidomide, and dexamethasone plus daratumumab; OS, overall survival; PFS, progression‐free survival; TTP, time to progression; VTd‐label, bortezomib/thalidomide/dexamethasone administered according to product labeling

Naïve, unadjusted comparisons between groups significantly favored VTd‐mod over VTd‐label for OS, PFS, and TTP, as well as D‐VTd versus VTd‐label (Figures 1 and 2 and Table 3). For response endpoints, naïve unadjusted comparisons found VTd‐mod to be inferior (≥CR) or noninferior (≥VGPR, ORR) to VTd‐label postinduction, whereas posttransplant responses for VTd‐mod were either inferior (≥CR) or superior (≥VGPR, ORR) to VTd‐label (Table 4). Similar results were observed for naïve unadjusted comparisons of D‐VTd with VTd‐label, with the exception of postinduction ≥VGPR and ORR, which were superior with D‐VTd versus VTd‐label (Table 4).

**TABLE 3:** Comparison of time‐to‐event endpoints for VTd‐mod vs VTd‐label and D‐VTd vs VTd‐label

		VTd‐mod vs VTd‐label		D‐VTd‐vs VTd‐label	
Endpoint	Comparison	HR (95% CI)	Wald test, *P*‐value^a^	HR (95% CI)	Wald test, *P*‐value^a^
OS	Naïve comparison	0.54 (0.306‐0.964)	**.037** **Superior**	0.21 (0.105‐0.423)	**<.0001** **Superior**
	On matched sample, primary analysis	0.60 (0.292‐1.220)	.157 Noninferior	0.16 (0.053‐0.489)	**.001** **Superior**
	On matched sample, sensitivity analysis	0.60 (0.259‐1.403)	.240 Inconclusive	0.28 (0.106‐0.748)	**.011** **Superior**
PFS	Naïve comparison	0.66 (0.460‐0.939)	**.021** **Superior**	0.29 (0.191‐0.435)	**<.0001** **Superior**
	On matched sample, primary analysis	0.84 (0.533‐1.327)	.456 Noninferior	0.30 (0.174‐0.528)	**<.0001** **Superior**
	On matched sample, sensitivity analysis	0.45 (0.254‐0.806)	.007 **Superior**	0.34 (0.176‐0.661)	**.001** **Superior**
TTP	Naïve comparison	0.61 (0.420‐0.895)	**.011** **Superior**	0.30 (0.194‐0.456)	**<.0001** **Superior**
	On matched sample, primary analysis	0.77 (0.473‐1.254)	.293 Noninferior	0.32 (0.182‐0.575)	**<.0001** **Superior**
	On matched sample, sensitivity analysis	0.44 (0.230‐0.822)	**.010** **Superior**	0.43 (0.251‐0.845)	**.015** **Superior**

Superior values are shown in bold font; stratified COX models were fitted as recommended by Austin PC, *J Thoracic Cardiovasc Surg* 2007; 134:1128‐35.

Abbreviations: CI, confidence interval; D, daratumumab; HR, hazard ratio; OS, overall survival; PFS, progression‐free survival; TTP, time to progression; VTd, bortezomib, thalidomide, and dexamethasone; VTd‐label, bortezomib/thalidomide/dexamethasone administered according to product labeling; *VTd‐mod*, bortezomib/thalidomide /dexamethasone modified dose.

^a^ The Wald test tests the significance of one or more independent variables in a regression.

**TABLE 4:** Comparison of response endpoints for VTd‐mod vs VTd‐label and D‐VTd vs VTd‐label

					VTd‐mod vs VTd‐label		D‐VTd vs VTd‐label	
Endpoint	Comparison	VTd‐mod, %	D‐VTd, %	VTd‐label, %	Rate difference (95% CI)	*P*‐value^a^	Rate difference (95% CI)	*P*‐value^a^
Postinduction ≥CR	Naïve comparison	8.9	14.4	35.4	−26.5 (−35.09 to −17.97)	**<.0001** **Inferior**	−21.0 (−29.75 to −12.29)	**<.0001** **Inferior**
	On matched sample, primary analysis	7.2	15.6	35.2	−28.0 (−36.96 to −19.04)	**<.0001** **Inferior**	−19.6 (−29.10 to −10.10)	**<.0001** **Inferior**
	On matched sample, sensitivity analysis	5.1	11.5	29.5	−24.4 (−35.05 to −13.66)	**<.0001** **Inferior**	−17.9 (−29.24 to −6.66)	**.0008** **Inferior**
Postinduction ≥VGPR	Naïve comparison	56.1	64.8	49.2	6.9 (−2.70 to 16.41)	.158 Noninferior	15.6 (6.11 to 25.08)	**.0010** **Superior**
	On matched sample, primary analysis	57.2	66.4	48.8	8.4 (−2.30 to 19.10)	.097 Noninferior	17.6 (7.06 to 28.14)	**.0009** **Superior**
	On matched sample, sensitivity analysis	50.0	59.6	44.9	5.1 (−8.41 to 18.67)	.469 Noninferior	14.7 (1.29 to 28.20)	**.019** **Superior**
Postinduction ORR	Naïve comparison	89.9	92.6	84.6	5.2 (−1.47 to 11.94)	0.089 Noninferior	8.0 (1.44 to 14.6)	**.0040** **Superior**
	On matched sample, primary analysis	88.0	92.0	84.8	3.2 (−4.27 to 10.67)	.388 Noninferior	7.2 (0.06 to 14.34)	**.024** **Superior**
	On matched sample, sensitivity analysis	86.5	91.0	85.9	0.6 (−8.76 to 10.04)	.897 Noninferior	5.1 (−3.80 to 14.06)	.238 Noninferior
Posttransplant ≥CR	Naïve comparison	14.6	22.7	46.9	−32.3 (−41.43 to −23.27)	**< .0001** **Inferior**	−24.3 (−33.54 to −15.00)	**< .0001** **Inferior**
	On matched sample, primary analysis	12.4	22.4	46.4	−34.0 (−43.65 to −24.35)	**<.0001** **Inferior**	−24.0 (−34.16 to −13.84)	**<.0001** **Inferior**
	On matched sample, sensitivity analysis	9.0	21.8	42.3	−33.3 (−45.18 to −21.49)	**<.0001** **Inferior**	−20.5 (−33.25 to −7.78)	**.0006** **Inferior**
Posttransplant ≥VGPR	Naïve comparison	67.3	76.8	55.4	12.0 (2.55 to 21.37)	**.010** **Superior**	21.4 (12.16 to 30.66)	**<.0001** **Superior**
	On matched sample, primary analysis	66.0	77.6	55.2	10.8 (0.29 to 21.31)	**.031** **Superior**	22.4 (12.27 to 32.53)	**<.0001** **Superior**
	On matched sample, sensitivity analysis	59.6	73.7	52.6	7.0 (−6.44 to 20.55)	.311 Noninferior	21.1 (8.10 to 34.21)	**.0006** **Superior**
Posttransplant ORR	Naïve comparison	90.4	92.3	77.7	12.7 (5.14 to 20.29)	**.0001** **Superior**	14.6 (7.07 to 22.07)	**<.0001** **Superior**
	On matched sample, primary analysis	89.6	91.6	78.4	11.2 (3.05 to 19.35)	**.004** **Superior**	13.2 (5.21 to 21.19)	**.0003** **Superior**
	On matched sample, sensitivity analysis	85.9	90.4	74.4	11.5 (0.41 to 22.66)	**.039** **Superior**	16.0 (5.29 to 26.76)	**.0015** **Superior**

Values in bold indicate statistically significant results.

Abbreviations: CI, confidence interval; CR, complete response; D, daratumumab; ORR, overall response rate; VGPR, very good partial response; VTd, bortezomib, thalidomide, and dexamethasone; VTd‐lab*e*l, bortezomib/thalidomide/dexamethasone administered according to product labeling; VTd‐mod, bortezomib/thalidomide/dexamethasone modified dose.

^a^ *P*‐value obtained from chi‐square test.

#### 3.2.1 VTd‐mod versus VTd‐label

**FIGURE 3:**
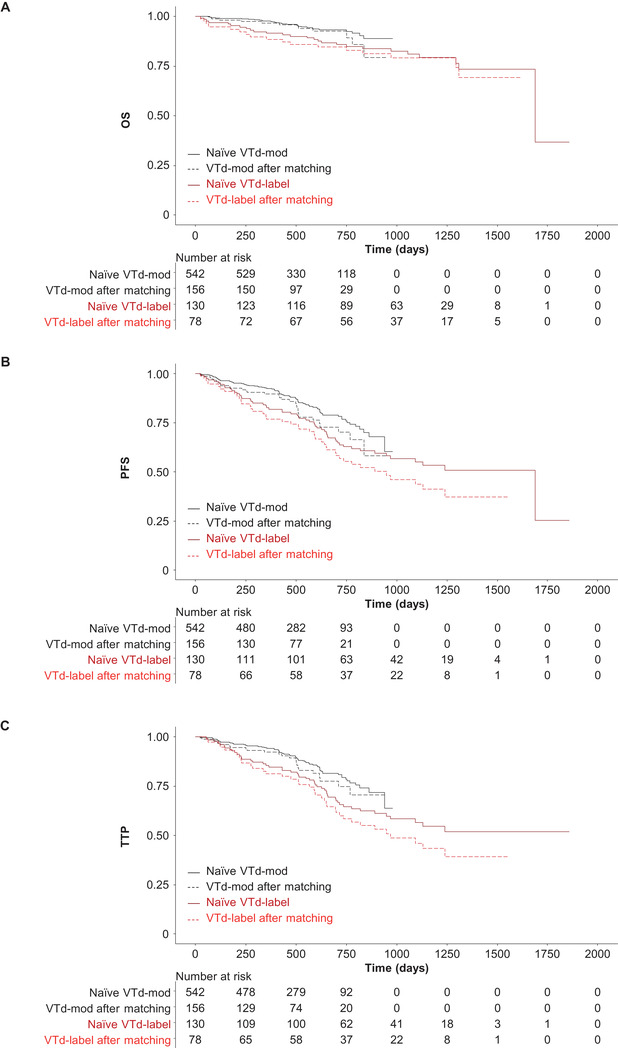
Sensitivity analysis for comparisons of (A) OS, (B) PFS, and (C) TTP for VTd‐mod vs VTd‐label. Abbreviations: OS, overall survival; PFS, progression‐free survival; TTP, time to progression; VTd‐label, bortezomib/thalidomide/dexamethasone administered according to product labeling; VTd‐mod, bortezomib/thalidomide/dexamethasone modified dose

After matching, OS (HR 0.60; 95% CI: 0.292‐1.220; *P* = .157), PFS (HR 0.84; 95% CI: 0.533‐1.327; *P* = .456), and TTP (HR 0.77; 95% CI: 0.473‐1.254; *P* = .293) for VTd‐mod were noninferior to VTd‐label for the primary analyses (Figure 1 and Table 3). For OS, the sensitivity analysis (which also included cytogenetic risk as a covariate) was inconclusive, although it should be noted that the sensitivity analysis was conducted on a reduced sample, as patients without cytogenetic testing were dropped from the study. The sensitivity analysis also suggested that PFS (HR 0.45; 95% CI: 0.254‐0.806; *P* = .007) and TTP (HR 0.44; 95% CI: 0.230‐0.822; *P* = .010) for VTd‐mod were significantly superior to VTd‐label (Table 3 and Figure 3).

For postinduction, the comparison on matched samples demonstrated that VTd‐mod was noninferior to VTd‐label for ORR and ≥VGPR postinduction, with similar proportions of patients demonstrating ORR (88.0% vs 84.8%; rate difference, 3.2 [95% CI: −4.27 to 10.67]; *P *= .388) and ≥VGPR (57.2% vs 48.8%; rate difference, 8.4 [95% CI: −2.30 to 19.10]; *P *= .097). Conversely, VTd‐mod was inferior to VTd‐label for ≥CR, with significantly fewer VTd‐mod patients exhibiting ≥CR compared with VTd‐label (7.2% vs 35.2%, respectively; rate difference, −28.0 [95% CI: −36.96 to −19.04]; *P *< .0001) (Table 4). Sensitivity analyses supported the primary analyses (Table 4).

When posttransplant responses were assessed after matching, ORR for VTd‐mod was significantly superior to VTd‐label (89.6% vs 78.4%; rate difference, 11.2 [95% CI: 3.05‐19.35]; *P *= .004) (Table 4). Additionally, VTd‐mod was significantly superior to VTd‐label for posttransplant ≥VGPR (66.0% vs 55.2%; rate difference, 10.8 [95% CI: 0.29‐21.31]; *P *= .031). However, in the sensitivity analysis, VTd‐mod was noninferior to VTd‐label (59.6% vs 52.6%; rate difference, 7.0 [95% CI: −6.44 to 20.55]; *P *= .311). VTd‐mod was inferior to VTd‐label for the proportion of patients with ≥CR (12.4% vs 46.4%; rate difference, −34.0 [95% CI: −43.65 to −24.35]; *P *< .0001) (**Table** **4**).

#### 3.2.2 D‐VTd versus VTd‐label

**FIGURE 4:**
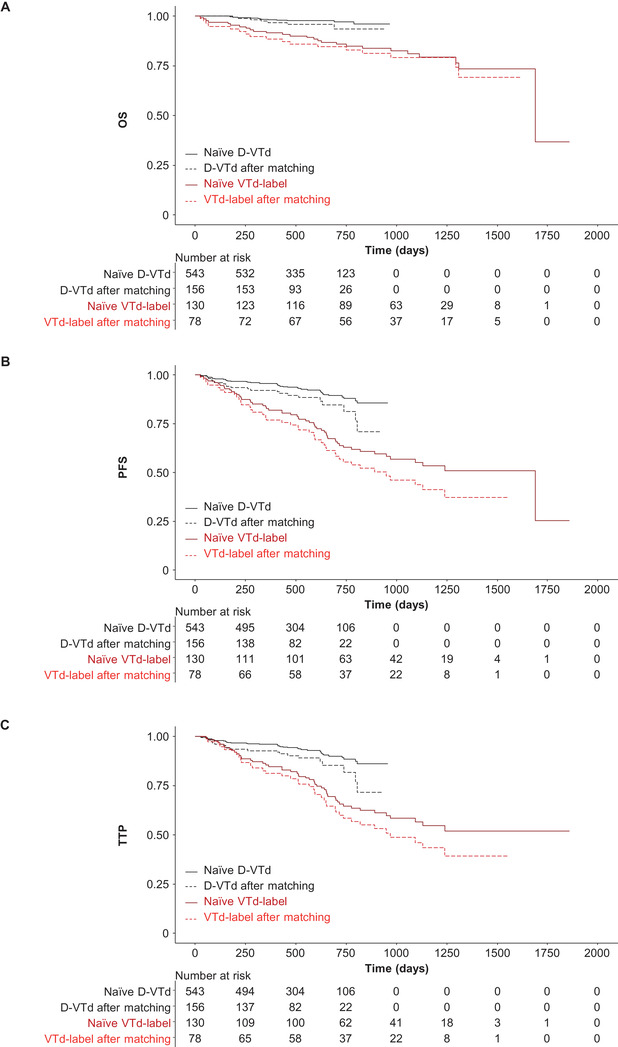
Sensitivity analysis for comparisons of (A) OS, (B) PFS, and (C) TTP for D‐VTd vs VTd‐label. Abbreviations: D‐VTd, bortezomib, thalidomide, and dexamethasone plus daratumumab; OS, overall survival; PFS, progression‐free survival; TTP, time to progression; VTd‐label, bortezomib/ thalidomide/dexamethasone administered according to product labeling

After matching, D‐VTd was significantly better than VTd‐label for OS (HR 0.16; 95% CI: 0.053‐0.489; *P* = .001), PFS (HR 0.30; 95% CI: 0.174‐0.528; *P* < .0001), and TTP (HR 0.32; 95% CI: 0.182‐0.575; *P* < .0001) (Figure 2 and Table 3). The sensitivity analysis supported the primary analysis for all time‐to‐event endpoints (Table 3 and Figure 4).

For response endpoints, D‐VTd was superior to VTd‐label for postinduction ≥VGPR (66.4% vs 48.8%; rate difference, 17.6 [95% CI: 7.06‐28.14]; *P *= .0009) and ORR (92.0% vs 84.8%; rate difference, 7.2 [95% CI: 0.06‐14.34; *P *= .024). However, in the sensitivity analysis for postinduction ORR, D‐VTd was noninferior to VTd‐label (91.0% vs 85.9%; rate difference, 5.1 [95% CI: −3.80 to 14.06]; *P *> .05) (Table 4). Conversely, D‐VTd was inferior to VTd‐label for postinduction ≥CR (15.6% vs 35.2%; rate difference, −19.6 [95% CI: −29.10 to −10.10]; *P *< .0001).

D‐VTd was significantly superior to VTd‐label for posttransplant ≥VGPR (77.6% vs 55.2%; rate difference, 22.4 [95% CI: 12.27‐32.53]; *P *< .0001) and ORR (91.6% vs 78.4%; rate difference, 13.2 [95% CI: 5.21‐21.19]; *P *= .0003) (Table 4). However, D‐VTd was significantly inferior to VTd‐label for posttransplant ≥CR after matching (22.4% vs 46.4%; rate difference, −24.0 [95% CI: −34.16 to −13.84]; *P *< .0001).

### 3.3 Safety

Baseline characteristics for the safety analyses before and after matching VTd‐mod to VTd‐label, and D‐VTd to VTd‐label, are summarized in Additional files 3 and 4 (in the Supporting Information). As with the efficacy analyses, matching balanced the groups in terms of baseline variables.

#### 3.3.1 VTd‐mod versus VTd‐label

After matching, VTd‐mod was noninferior to VTd‐label for all evaluated safety endpoints, including the rate of discontinuation due to AEs (6.4% for both groups; *P *= 1.000), the rate of discontinuation due to grade 3 or 4 AEs (5.2% vs 3.2%; *P *= .377), and the incidence of grade 3 or 4 peripheral neuropathy (6.8% vs 5.6%; *P *= .658) (Table 5).

**TABLE 5:** Comparison of safety endpoints: VTd‐mod and D‐VTd versus VTd‐label

					VTd‐mod vs VTd‐label		D‐VTd vs VTd‐label	
Endpoint	Comparison	VTd‐mod, %	D‐VTd, %	VTd‐label, %	Rate difference (95% CI)	*P*‐value^a^	Rate difference (95% CI)	*P*‐value^a^
Treatment discontinuation due to AEs, all grades	Naïve comparison	5.4	5.2	6.2	−0.8 (−5.31 to 3.79)	.718 Noninferior	−0.9 (−5.47 to 3.61)	0.650 Noninferior
	On matched sample, primary analysis	6.4	5.6	6.4	0 (−5.26 to 5.26)	1.000 Noninferior	−0.8 (−5.95 to 4.35)	0.752 Noninferior
	On matched sample, sensitivity analysis	6.4	6.4	6.4	0 (−6.66 to 6.66)	1.000 Noninferior	0 (−6.66 to 6.66)	1.000 Noninferior
Treatment discontinuation due to AEs, grades 3 or 4	Naïve comparison	4.3	3.9	3.1	1.2 (−2.23 to 4.62)	.543 Noninferior	0.8 (−2.55 to 4.23)	.669 Noninferior
	On matched sample, primary analysis	5.2	4.0	3.2	2.0 (−2.13 to 6.13)	.377 Noninferior	0.8 (−3.13 to 4.73)	.695 Noninferior
	On matched sample, sensitivity analysis	5.1	4.5	2.6	2.6 (−2.36 to 7.49)	.371 Noninferior	1.9 (−2.86 to 6.7)	.453 Noninferior
Peripheral neuropathy, grade 3 or 4	Naïve comparison	6.3	3.7	5.4	0.9 (−3.46 to 5.33)	.704 Noninferior	−1.6 (−5.85 to 2.55)	.375 Noninferior
	On matched sample, primary analysis	6.8	2.8	5.6	1.2 (−3.90 to 6.30)	.658 Noninferior	−2.8 (−7.32 to 1.72)	.186 Noninferior
	On matched sample, sensitivity analysis	5.8	3.8	6.4	−0.6 (−7.19 to 5.91)	.838 Noninferior	−2.6 (−8.78 to 3.65)	.371 Noninferior

Abbreviations: AE, adverse event; CI, confidence interval; D, daratumumab; VTd, bortezomib, thalidomide, and dexamethasone; VTd‐label, bortezomib/thalidomide/dexamethasone administered according to product labeling; VTd‐mod, bortezomib/thalidomide/dexamethasone modified dose.

^a^ *P*‐value obtained from the chi‐square test.

#### 3.3.2 D‐VTd versus VTd‐label

For all evaluated safety endpoints, D‐VTd was noninferior to VTd‐label (Table 5). The rate of discontinuation due to AEs of all grades was 5.6% for D‐VTd versus 6.4% for VTd‐label (*P *= .752), whereas the rate of discontinuation due to grade 3 or 4 AEs was 4.0% versus 3.2%, respectively (*P *= .695), and the incidence of grade 3 or 4 peripheral neuropathy was 2.8% versus 5.6% (*P *= .186).

## 4 DISCUSSION

VTd, one of the standard‐of‐care regimens used in clinical practice for first‐line treatment of transplant‐eligible patients with NDMM, increasingly includes a modified (lower) fixed dose of thalidomide to reduce toxicity [7, 17, 18]. This modified regimen (VTd‐mod) was evaluated in the first part of the phase III CASSIOPEIA study, wherein patients with NDMM received pre‐ASCT induction and post‐ASCT consolidation therapy with VTd using a thalidomide dose of 100 mg/day, with or without daratumumab [7]. To date, there are no RCTs to directly compare the efficacy and safety of the VTd‐mod regimen versus the regimen using the original dose of thalidomide, per the product label (VTd‐label). When direct comparisons are not feasible, indirect comparisons can be performed using pharmacoepidemiologic methods such as PSM, which can create treatment groups with prognostic similarity. PSM methodology was used in the current analysis to estimate the comparative efficacy and safety of the modified VTd regimen (with or without daratumumab) versus VTd‐label. Balanced cohorts were formed by adjusting for cross‐study differences in baseline characteristics that might affect outcomes and treatment assignments. This method reduces the impact of confounding, thereby strengthening the validity and confidence of findings [19, 20].

In the current PSM analysis, the VTd‐mod regimen was found to be noninferior to VTd‐label for OS, PFS, TTP, postinduction ≥VGPR and ORR, and safety endpoints; inferior to VTd‐label for postinduction and posttransplant ≥CR; and significantly better than VTd‐label for posttransplant ≥VGPR and ORR. The finding that the modified regimen did not have a superior safety profile to the regimen with the higher dose (VTd‐label) was somewhat unexpected. One possible explanation for these results is that the CASSIOPEIA and PETHEMA/GEM studies were performed at different times in different centers with varying geographic locations, which could be associated with potential patient selection bias as well as different efficacy and safety reporting standards that could have affected the results. For example, reporting rules on safety in the PETHEMA/GEM study may not have been as rigorous as those in CASSIOPEIA. The observed rates of grade 3 or 4 treatment‐emergent hematologic toxicities reported in PETHEMA/GEM were much lower than the rates reported in CASSIOPEIA [7, 10, 11]. When laboratory values, which are more objective, were used to estimate hematologic toxicities instead of physician‐reported events, the rate of grade 3 or 4 treatment‐emergent hematologic toxicities appears much higher in the PETHEMA/GEM study than the physician‐reported grade 3 or 4 AEs would indicate; laboratory values appear not to have been reported as AEs unless patients experienced symptoms. Thus, there was a reporting bias towards the null for hematologic toxicities in the PETHEMA/GEM study.

In terms of efficacy, response criteria in CASSIOPEIA were more stringent than in the PETHEMA/GEM study, which may have resulted in an underestimation of the benefit of VTd‐mod compared with VTd‐label (and may thus explain the noninferior to inferior efficacy results on time‐to‐event endpoints and ≥CR). Response in PETHEMA/GEM was investigator assessed, whereas response in CASSIOPEIA was determined by a strict computer algorithm. Also, the PETHEMA/GEM study applied European Society for Blood and Marrow Transplantation criteria to assess response [10], whereas CASSIOPEIA used International Myeloma Working Group criteria [7], which are more rigorous. Despite this, posttransplant ≥VGPR and ORR with the reduced dose of thalidomide were superior to results with VTd‐label. As the criteria for VGPR and ORR are less stringent than CR criteria, it is perhaps not surprising that superiority for VTd‐mod relative to VTd‐label was achieved in these categories, but the finding is positive nonetheless.

Our analysis also indicated that following PSM, the daratumumab‐containing regimen was associated with statistically significant improvements in OS, PFS, TTP, and postinduction and posttransplant ≥VGPR and ORR compared with VTd‐mod. These results are in agreement with those of the CASSIOPEIA trial, which demonstrated the clinical benefit of adding daratumumab to VTd in transplant‐eligible NDMM patients [7]. However, OS results for VTd‐mod and D‐VTd should be interpreted with the caveat that CASSIOPEIA is ongoing, so OS data remain immature (medians have not yet been reached). The current PSM analysis also found D‐VTd to be noninferior to VTd‐label for safety endpoints, which also agrees with the CASSIOPEIA trial results, demonstrating that the addition of daratumumab to VTd does not increase overall toxicity or affect the ability of patients to undergo successful transplantation [7].

Propensity score–based methods do have some limitations, which should also be considered when interpreting these findings. First, the PSM analysis could not be adjusted for unreported or unobserved confounding factors that may influence patient outcomes (residual confounding). If any important variables were omitted, then the groups may remain unbalanced and study results can be seriously biased [21]. However, both CASSIOPEIA and PETHEMA/GEM are RCTs, with data from most of the clinically relevant baseline variables collected and included in this analysis; therefore, the risk of unobserved confounding may be minimized. Second, PSM requires large samples because matching reduces the sample size, negatively affecting the precision of the estimates. PSM also cannot correct for selection bias, and regional differences in local standard‐of‐care regimens may have further contributed to differences in the CASSIOPEIA and PETHEMA/GEM studies. Lastly, a substantial number of patients without cytogenetic risk data in the PETHEMA/GEM study were excluded from the primary analysis, thereby reducing the power of the sensitivity analysis.

In addition to limitations of the PSM methodology, longer median follow‐up and differences in maintenance treatments between the CASSIOPEIA and PETHEMA/GEM studies may have biased the results in favor of the VTd‐label arm, particularly for long‐term survival. Median follow‐up in PETHEMA/GEM for VTd‐label was 35.2 months [10, 11] compared with 18.8 months in CASSIOPEIA [7]. Thus, more patients in the VTd‐label arm of the analysis were exposed to maintenance therapies compared with VTd‐mod/D‐VTd. Fewer patients in the CASSIOPEIA study had the opportunity to receive maintenance treatment because, per protocol for Part 2 of the study, patients with partial response or better were rerandomized 100 days post‐ASCT in a 1:1 ratio to either observation only or daratumumab monotherapy every 8 weeks for a maximum of 2 years; thus, 50% of patients did not receive maintenance treatment. In PETHEMA/GEM, patients were rerandomized 3 months post‐ASCT in a 1:1:1 ratio to one of three maintenance therapies. Comparisons may also be biased in favor of VTd‐label due to differences in how response was assessed between the studies.

## 5 CONCLUSIONS

This PSM analysis demonstrated the noninferiority of VTd‐mod versus VTd‐label for OS, PFS, TTP, and postinduction ≥VGPR and ORR, and posttransplant superiority of VTd‐mod for ≥VGPR and ORR. Safety outcomes for VTd‐mod were also noninferior to VTd‐label outcomes despite a lower dose of thalidomide in the VTd‐mod regimen. In addition, D‐VTd, using a modified dose of thalidomide 100 mg, was significantly better than VTd‐label for efficacy outcomes (OS, PFS, TTP, and postinduction and posttransplant ≥VGPR and ORR) and was noninferior to VTd‐label for safety outcomes. Taken together, these findings confirm those of Part 1 of the CASSIOPEIA study wherein D‐VTd had superior efficacy to VTd, with both regimens using a modified thalidomide dose, and support the use of daratumumab in the first‐line treatment for NDMM in patients who are transplant‐eligible.

## AUTHOR CONTRIBUTIONS

PM, CH, SZ, and PS contributed to the accrual and treatment of patients, data acquisition, interpretation and analysis of data from the CASSIOPEIA study. CB, VV, TK, JH, AL, and SC contributed to the study concept, designed the PSM analysis, and interpreted the data. MH, YH, and BH performed the PSM analyses. All authors participated in manuscript preparation and revisions, and all authors read and approved the final manuscript.

## CONFLICT OF INTERESTS

PM has been a consultant to and received honoraria from AbbVie, Amgen, Celgene, Janssen, and Takeda. CH has been a consultant to Celgene and has received honoraria from AbbVie, Amgen, Celgene, and Janssen. SZ has been a member of an entity's board of directors or advisory committees and received research funding from Celgene, Janssen Pharmaceuticals, and Takeda. MH and BH are employees of Ingress‐Health. YH has been a consultant to and been employed by Ingress‐Health, and has received funding from Pfizer. PS has received honoraria and research funding from Amgen, Celgene, Janssen, Karyopharm, and Takeda and has received honoraria from BMS and research funding from SkylineDx. CdB, VV, TK, JH, AL, and SC are employees of Janssen.

6

## Supporting information

Additional file 1. Figure: Summary of CASSIOPEIA and PETHEMA/GEM study designs

Additional file 2. Table: Summary of efficacy outcomes for CASSIOPEIA and PETHEMA/GEM

Additional file 3. Table: Key baseline characteristics for VTd‐mod (CASSIOPEIA) and VTd‐label (PETHEMA/GEM) — safety analyses, pre‐ and post‐matching

Additional file 4. Table: Key baseline characteristics for D‐VTd (CASSIOPEIA) and VTd‐label (PETHEMA/GEM) — safety analyses, pre‐ and post‐matching

## Data Availability

The data sharing policy of Janssen Pharmaceutical Companies of Johnson & Johnson is available at https://www.janssen.com/clinical-trials/transparency. As noted on that site, requests for access to the study data can be submitted through the Yale Open Data Access (YODA) Project site at http://yoda.yale.edu.
