# Supplementary material for: Comparative efficacy and safety of bortezomib, thalidomide, and dexamethasone (VTd) without and with daratumumab (D‐VTd) in CASSIOPEIA versus VTd in PETHEMA/GEM in transplant‐eligible patients with newly diagnosed multiple myeloma, using propensity score matching
Source: EJHaem. 2020 Nov 7;2(1):66–80. doi: 10.1002/jha2.129 (PMC9175692; doi:10.1002/jha2.129)
Supplement: Supplementary file 1 — Additional file 1. Figure: Summary of CASSIOPEIA and PETHEMA/GEM study designs [file JHA2-2-66-s002.docx]

**Additional file 1**. **Summary of the CASSIOPEIA and PETHEMA/GEM study designs**


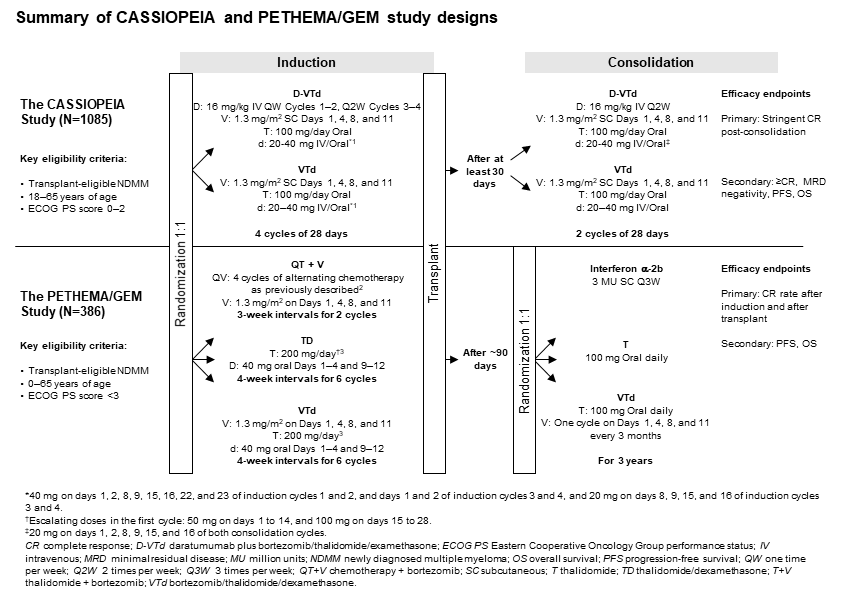


**References**

1. Moreau P, Attal M, Hulin C, Arnulf B, Belhadj K, Benboubker L, et al. Bortezomib, thalidomide, and dexamethasone with or without daratumumab before and after autologous stem-cell transplantation for newly diagnosed multiple myeloma (CASSIOPEIA): a randomised, open-label, phase 3 study. Lancet. 2019;394:29-38. doi: 10.1016/S0140-6736(19)31240-1.
2. Rosiñol L, Oriol A, Teruel AI, Hernández D, López-Jiménez J, de la Rubia J, et al. Superiority of bortezomib, thalidomide, and dexamethasone (VTD) as induction pretransplantation therapy in multiple myeloma: a randomized phase 3 PETHEMA/GEM study. Blood. 2012;120:1589-96. doi: 10.1182/blood-2012-02-408922.
3. Rosiñol L, Oriol A, Teruel AI, de la Guía AL, Blanchard M, de la Rubia J, et al. Bortezomib and thalidomide maintenance after stem cell transplantation for multiple myeloma: a PETHEMA/GEM trial. Leukemia. 2017;31:1922-7. doi: 10.1038/leu.2017.35.
