# Supplementary material for: Comparative efficacy and safety of bortezomib, thalidomide, and dexamethasone (VTd) without and with daratumumab (D‐VTd) in CASSIOPEIA versus VTd in PETHEMA/GEM in transplant‐eligible patients with newly diagnosed multiple myeloma, using propensity score matching
Source: EJHaem. 2020 Nov 7;2(1):66–80. doi: 10.1002/jha2.129 (PMC9175692; doi:10.1002/jha2.129)
Supplement: Supplementary file 2 — Additional file 2. Table: Summary of efficacy outcomes for CASSIOPEIA and PETHEMA/GEM [file JHA2-2-66-s004.docx]

**Additional file 2.** Summary of efficacy outcomes for CASSIOPEIA and PETHEMA/GEM

| **Efficacy Outcome** | **CASSIOPEIA [1]** | | **PETHEMA/GEM [2]** | | |
| --- | --- | --- | --- | --- | --- |
|  | **D-VTd**  **(n = 543)** | **VTd-mod**  **(n = 542)** | **QT+V**  **(n = 129)** | **TD**  **(n = 127)** | **VTd-label**  **(n = 130)** |
| Post-induction ≥CR, % | 14 | 9 | 21** | 14** | 35** |
| Post-transplant ≥CR, % | 39* | 26* | 38 | 24 | 46 |
| MRD negativity, any response, 10^-5^ | 64* | 44* | NA | NA | NA |
| PFS, median, months | NR (ongoing)^†^ | NR (ongoing)^†^ | 35.3^‡^ | 28.2^‡^ | 56.2^‡^ |
| OS, % | NR (ongoing)^†^ | NR (ongoing)^†^ | 70^§^ | 65^§^ | 74^§^ |

CR, complete response; D-VTd, daratumumab-VTd; MRD, minimal residual disease; NA, not available; NR, not reached; OS, overall survival; PFS, progression-free survival; QT+V, chemotherapy + bortezomib; TD, thalidomide/dexamethasone; VTd-label, bortezomib/thalidomide / dexamethasone administered according to product labeling; VTd-mod, bortezomib/thalidomide /dexamethasone modified dose.

**P* < ∙0001; **VTD vs QT+V, *P* = .01; VTD vs TD, *P* = .001;

^†^After 33 months.

^‡^After median follow-up of 35.2 months.

^§^After 4 years of post-randomization.

**References**

# Moreau P, Attal M, Hulin C, Arnulf B, Belhadj K, Benboubker L, et al. Bortezomib, thalidomide, and dexamethasone with or without daratumumab before and after autologous stem-cell transplantation for newly diagnosed multiple myeloma (CASSIOPEIA): a randomised, open-label, phase 3 study. Lancet 2019;394(10192):29-38. doi: 10.1016/S0140-6736(19)31240-1.

1. Rosiñol L, Oriol A, Teruel AI, Hernández D, López-Jiménez J, de la Rubia J, et al. Superiority of bortezomib, thalidomide, and dexamethasone (VTD) as induction pretransplantation therapy in multiple myeloma: a randomized phase 3 PETHEMA/GEM study. Blood. 2012;120:1589-96. doi: 10.1182/blood-2012-02-408922.
