# Supplementary material for: Comparative efficacy and safety of bortezomib, thalidomide, and dexamethasone (VTd) without and with daratumumab (D‐VTd) in CASSIOPEIA versus VTd in PETHEMA/GEM in transplant‐eligible patients with newly diagnosed multiple myeloma, using propensity score matching
Source: EJHaem. 2020 Nov 7;2(1):66–80. doi: 10.1002/jha2.129 (PMC9175692; doi:10.1002/jha2.129)
Supplement: Supplementary file 3 — Additional file 3. Table: Key baseline characteristics for VTd‐mod (CASSIOPEIA) and VTd‐label (PETHEMA/GEM) — safety analyses, pre‐ and post‐matching [file JHA2-2-66-s003.docx]

**Additional file 3.** Key baseline characteristics for VTd-mod (CASSIOPEIA) and VTd-label (PETHEMA/GEM) — safety analyses, pre- and post-matching

|  | |  | | | | | **Matched patient population** | | | | | | |
| --- | --- | --- | --- | --- | --- | --- | --- | --- | --- | --- | --- | --- | --- |
| **Variables** | | **Unmatched patient population** | | | | | **Primary analysis** | | | | **Sensitivity analysis** | | |
|  | **VTd-mod, CASSIOPEIA** | | **VTd-label, PETHEMA/ GEM** | | **Absolute standardized difference  pre-match** | | **VTd-mod, CASSIOPEIA** | **VTd-label, PETHEMA/ GEM** | | **Absolute standardized difference post-match** | **VTd-mod, CASSIOPEIA** | **VTd-label, PETHEMA/ GEM** | **Absolute standardized difference post-match** |
| Sample size, *n* | | 538 | 130 | | NA | | 250 | 125 | | NA | 156 | 78 | NA |
| Age, mean, y | | 56.5 | 55.6 | | **0.118** | | 55.6 | 55.4 | | 0.017 | 55.0 | 54.9 | 0.012 |
| Male, % | | 58.9 | 58.5 | | 0.009 | | 60.8 | 57.6 | | 0.065 | 56.4 | 59.0 | 0.052 |
| ECOG PS ≥1, % | | 52.2 | 56.2 | | 0.079 | | 52.8 | 54.4 | | 0.032 | 55.8 | 57.7 | 0.039 |
| IgG myeloma, % | | 61.3 | 66.2 | | 0.100 | | 68.8 | 66.4 | | 0.051 | 66.7 | 69.2 | 0.055 |
| ISS staging, % | |  |  | |  | |  |  | |  |  |  |  |
| ISS I | | 42.0 | 33.9 | | **0.215** | | 32.0 | 34.4 | | 0.081 | 30.8 | 32.1 | 0.030 |
| ISS II | | 42.9 | 43.9 | | NA | | 46.4 | 42.4 | | NA | 46.2 | 44.9 | NA |
| CL_CR_, mean mL/min | | 100.0 | 86.47 | | **0.386** | | 89.0 | 86.8 | | 0.070 | 88.5 | 89.4 | 0.029 |
| Hemoglobin level, mean, g/L | | 114.8 | 111.2 | | **0.203** | | 111.5 | 111.5 | | 0.003 | 108.8 | 109.6 | 0.050 |
| Platelet count, mean, x10^9^/L | | 252.8 | 235.9 | | **0.191** | | 236.1 | 235.9 | | 0.003 | 247.9 | 242.2 | 0.059 |
| Cytogenetic risk, % | |  | |  | |  |  | |  |  |  |  |  |
| Testing not done | | 0.4 | | 36.9 | | **1.067** | Not included in Primary Analysis | | | | NA* | NA* | NA* |
| High risk | | 16.0 | | 12.3 | | NA |  |  |  |  | 18.0 | 19.2 | 0.033 |
| Standard risk | | 83.6 | | 50.8 | | NA |  |  |  |  | 82.1 | 80.8 | NA |

Standardized mean differences >0.1 suggest potentially important imbalances (**indicated in bold**).

CL_CR_, creatinine clearance; ECOG PS, Eastern Cooperative Oncology Group performance status; IgG, immunoglobulin G; ISS, Multiple Myeloma International Staging System; VTd bortezomib, thalidomide, and dexamethasone.

*In the Sensitivity Analysis, patients with no cytogenetic test done were excluded from the dataset.
