# Supplementary material for: Comparative efficacy and safety of bortezomib, thalidomide, and dexamethasone (VTd) without and with daratumumab (D‐VTd) in CASSIOPEIA versus VTd in PETHEMA/GEM in transplant‐eligible patients with newly diagnosed multiple myeloma, using propensity score matching
Source: EJHaem. 2020 Nov 7;2(1):66–80. doi: 10.1002/jha2.129 (PMC9175692; doi:10.1002/jha2.129)
Supplement: Supplementary file 4 — Additional file 4. Table: Key baseline characteristics for D‐VTd (CASSIOPEIA) and VTd‐label (PETHEMA/GEM) — safety analyses, pre‐ and post‐matching [file JHA2-2-66-s001.docx]

**Additional file 4.** Key baseline characteristics for D-VTd (CASSIOPEIA) and VTd-label (PETHEMA/GEM) — safety analyses, pre- and post-matching

|  |  | | | **Matched patient population** | | | | | |
| --- | --- | --- | --- | --- | --- | --- | --- | --- | --- |
| **Variables** | **Unmatched patient population** | | | **Primary analysis** | | | **Sensitivity analysis** | | |
|  | **D-VTd, CASSIOPEIA** | **VTd-label, PETHEMA/GEM** | **Absolute standardized difference pre-match** | **D-VTd, CASSIOPEIA** | **VTd-label, PETHEMA/GEM** | **Absolute standardized difference post-match** | **D-VTd, CASSIOPEIA** | **VTd-label, PETHEMA/GEM** | **Absolute standardized difference post-match** |
| Sample size, *n* | 536 | 130 | NA | 250 | 125 | NA | 156 | 78 | NA |
| Age, mean, y | 56.8 | 55.6 | **0.171** | 55.0 | 55.4 | 0.060 | 54.9 | 54.9 | 0.002 |
| Male, % | 58.4 | 58.5 | 0.001 | 60.8 | 57.6 | 0.065 | 59.6 | 59.0 | 0.013 |
| ECOG PS ≥1, % | 51.3 | 56.2 | 0.097 | 53.2 | 54.4 | 0.024 | 59.6 | 57.7 | 0.039 |
| IgG myeloma, % | 64.4 | 66.2 | 0.038 | 68.0 | 66.4 | 0.034 | 65.4 | 69.2 | 0.082 |
| ISS staging, % |  |  |  |  |  |  |  |  |  |
| ISS I | 37.9 | 33.9 | **0.181** | 36.8 | 34.4 | 0.060 | 32.7 | 32.1 | 0.056 |
| ISS II | 46.8 | 43.9 | NA | 39.6 | 42.4 | NA | 42.3 | 44.9 | NA |
| CL_CR_, mean, mL/min | 103.6 | 86.5 | **0.360** | 90.0 | 86.8 | **0.102** | 89.8 | 89.4 | 0.013 |
| Hemoglobin level, mean g/L | 112.5 | 111.2 | 0.073 | 111.6 | 111.5 | 0.006 | 110.2 | 109.6 | 0.029 |
| Platelet count, mean, x10^9^/L | 248.8 | 235.9 | **0.139** | 237.7 | 235.9 | 0.020 | 242.6 | 242.2 | 0.004 |
| Cytogenetic risk, % |  |  |  |  |  |  |  |  |  |
| Testing not done | 0.2 | 36.9 | **1.081** | Not included in the Primary Analysis | | | NA | NA | NA |
| High risk | 14.9 | 12.3 | NA |  |  |  | 16.7 | 19.2 | 0.067 |
| Standard risk | 84.9 | 50.8 | NA |  |  |  | 83.3 | 80.8 | NA |

Standardized mean differences >0.1 suggest potentially important imbalances (**bold**).

CL_CR_, creatinine clearance; D, daratumumab; ECOG PS, Eastern Cooperative Oncology Group performance status; IgG, immunoglobulin G; ISS, Multiple Myeloma International Staging System; VTd, bortezomib, thalidomide, and dexamethasone.

*In the Sensitivity Analysis, patients with no cytogenetic test done were excluded from the dataset.
